# Case Report: Cetuximab and nivolumab use in advanced cutaneous squamous cell carcinoma resistant to chemotherapy

**DOI:** 10.12688/f1000research.19149.2

**Published:** 2019-12-31

**Authors:** Alvise Sernicola, Salvatore Lampitelli, Federica Marraffa, Patrizia Maddalena, Sara Grassi, Antonio Giovanni Richetta, Stefano Calvieri

**Affiliations:** 1 Unit of Dermatology, Sapienza University of Rome, Piazzale Aldo Moro 5, Rome, 00185, Italy

**Keywords:** cutaneous squamous cell carcinoma, cetuximab, EGFR, non-melanoma skin cancer

## Abstract

We present the case of a 60-year-old man with unresectable cutaneous squamous cell carcinoma (cSCC) of the sternal area, which was not amenable to radiation therapy (stage III, T3N0M0). The treatment history of this patient is remarkable as the disease had progressed through all lines of conventional therapy established in the literature. The patient was treated with epidermal growth factor receptor (EGFR) inhibitor cetuximab for 35 cycles and restaged after 12 months of therapy with a whole body CT scan, documenting stage IV disease (T3N2bM1). The use of cetuximab as a single agent was effective for a limited time and we decided to initiate combination therapy with cetuximab and nivolumab. Restaging after six months of this combination regimen documented stable disease.

## Introduction

This case describes the effective use of cetuximab and nivolumab in an extensive thoracic cutaneous squamous cell carcinoma resistant to all previous lines of chemotherapy.

Non-melanoma skin cancer (NMSC) is the most common malignant neoplasm affecting Caucasian individuals, the main types of which are basal cell carcinoma (BCC) and squamous cell carcinoma (SCC). SCC has a lower incidence than BCC and the gold standard of treatment is surgical excision. Between 1–5% of SCCs exhibit biologically aggressive behavior and are resistant to surgery ^1^.

The management of metastatic or locally advanced cutaneous SCC (cSCC), traditionally relying on conventional radiotherapy (alone or in combination with surgery) and systemic chemotherapy, benefited from the promising addition of targeted inhibitors of the epidermal growth factor receptor (EGFR) pathway and dramatically changed following the introduction of immunotherapy with checkpoint inhibitors ^2^. Anti-EGFR monoclonal antibody cetuximab, at the standard weekly dosage of 250mg/m2, provides an off-label treatment option with potential clinical value in advanced cSCC ^3^.

In 2018, a study by Migden *et al.* dramatically changed the previous scenario establishing the new standard of care with PD-1 blockade in immunocompetent patients, in the absence of contraindications to immunotherapy ^4^. Anti-PD1 monoclonal antibody cemiplimab was consequently approved for use in Europe in July 2019.

## Case presentation

**Figure 1.**
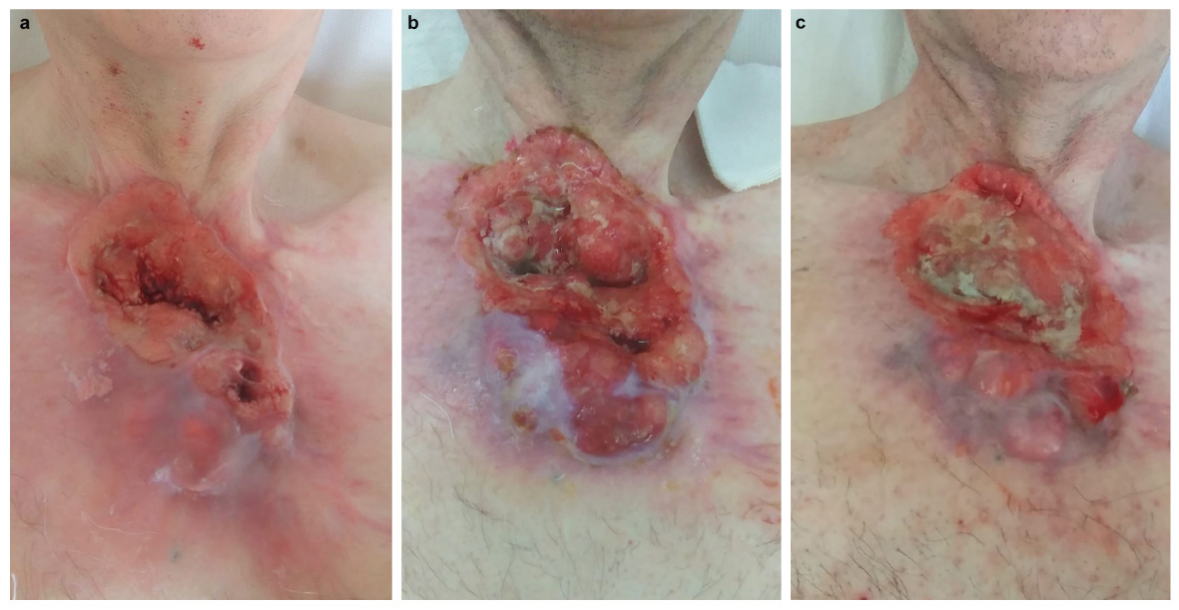
Clinical presentation before cetuximab ( **a**) and after six ( **b**) and 12 weeks of therapy ( **c**).

A 60-year-old Caucasian man, currently unemployed, presented to our dermatology department complaining of the recurrence of a thoracic cSCC. Physical examination revealed an extensive ulcerative skin lesion of the sternal area covered by necrotic and fibrinous tissue. The patient reported intermittent pain and bleeding ( Figure 1).

The onset of a nodular skin lesion in the same site dated back to 2000, but an initial diagnosis of BCC was made only in 2013, when a single biopsy was performed (see Table 1 for timeline). A computerized tomography (CT) scan followed, demonstrating a high local burden of disease, with destructive osteo-muscular infiltration, preventing a surgical or radiation approach, and the patient was treated with vismodegib (150 mg daily). After 12 months of apparent clinical remission, a local relapse was observed, and the histologic examination of an excisional biopsy diagnosed SCC. Surgical removal of the tumor was not radical, and the patient was referred for adjuvant chemotherapy, failing four consecutive cytotoxic regimens, until the personal decision of the patient to withdraw from treatment.

**Table 1.** Timeline of interventions and outcomes.

Timeline	Medical history and past interventions	
	No family history of skin cancer 1999: total gastrectomy for gastric adenocarcinoma	
	**Diagnostic testing and interventions**	
Past Interventions 2013 – 2017	2000: Patient reports onset of nodular skin lesion 2013: Incisional biopsy: BCC CT scan (23-Jan-2013): high local burden of disease (50mm AP diameter), with destructive osteo-muscular infiltration - *vismodegib* 150mg daily from Feb to Nov-2013 2014: relapse of nodular skin lesion Excision biopsy (Feb-2014): SCC Wide surgical excision (22-May-2014): not radical Adjuvant chemotherapy: - *cisplatinum* 100mg/m2 day 1 with *fluorouracil* 1000mg/m2 for four days of 21-day-cycles, Aug – Sep-2014 - *radio-chemotherapy with gemcitabine* Dec-2014 – Jan-2015 - *cisplatinum* 100mg/m2 day 1 with *docetaxel* 75mg/m2 day 1 of 21-day-cycles Aug-2016 – Nov-2016 - *gemcitabine monotherapy* 3000mg/m2 on day 1 and 15 of 28-day-cycles Dec-2016 – Jul-2017	
31-Jan-2018	Baseline assessment stage III T3N0M0, ECOG 0	Immunohistochemistry: low/no PD-L1 expression CT scan (31-Jan-2018): DT×DAP×DL 120×62×110mm
19-Apr-2018	Cetuximab monotherapy 35 cycles	- *cetuximab* initial single dose of 400mg/m2 followed by - *cetuximab* 250mg/m2 weekly for seven cycles followed by - *cetuximab* 250mg/m2 every two weeks CT scan (28-May-2018): DAP 72mm CT scan (10-Sep-2018): DT×DAP×DL 135×70×110mm CT scan (12-Dec-2018): DT×DAP×DL 153×70×120mm
20-Feb-2019	Restaging stage IV T3N2bM1 ECOG 1	CT scan (20-Feb-2019) unchanged dimension, development of lymphadenopathies, the greater of which in the right paratracheal region DT 16mm and in the right supra- and sub-clavicular region DT 15mm, right axillary lymph node 6×6mm, development of secondary osteolytic lesions of D8 and D9 vertebral bodies
22-May-2019	Cetuximab/nivolumab combination therapy 13 cycles of each agent	- *cetuximab* single dose of 250mg/m2 Q2W and *nivolumab* single fixed dose of 240mg Q2W administered at alternating weeks CT scan (13-May-2019) unchanged dimensions (DAP 60mm), slight reduction of previous lymphadenopathies DM 13mm and DM 10mm respectively, increased right axillary lymph node 10×8mm, stable secondary lesions of D8 and D9 vertebral bodies with marked reduction of pre- and paravertebral tissue involvement compared to previous exam CT scan (06-Aug-2019) slight dimensional increase with DL×DT×DAP 59×43×67mm
18-Nov-2019	Restaging stage IV T3N2bM1 ECOG 1	CT scan (18-Nov-2019) DL×DT×DAP 78×60×85mm, unchanged lymphadenopathies DM 13mm and DM 10mm respectively, stable secondary lesions of D8 and D9 vertebral bodies

[i] BCC, basal cell carcinoma; CT, computerized tomography; DAP, anterior-posterior diameter; DL, longitudinal diameter; DM, maximum diameter; DT, transverse diameter; PDL-1, programmed cell death ligand-1; SCC, squamous cell carcinoma.

**Figure 2.**
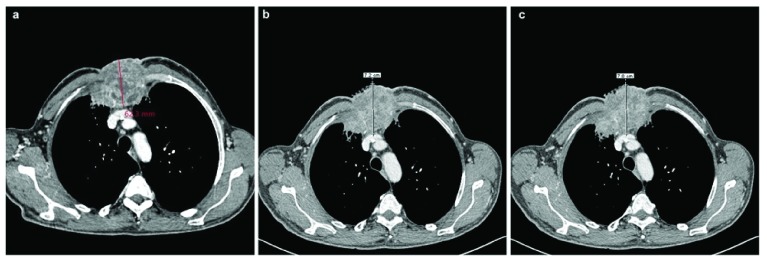
CT scan performed at baseline ( **a**), after six ( **b**) and 12 weeks of therapy ( **c**), highlighting the anterior-posterior diameter of the tumor.

A stage III-disease (T3N0M0, Figure 2a) ^5^ advised the use of anti-PD-1 therapy. Even if PDL-1 testing is not required, immunohistochemistry was performed on the previous biopsy sample documenting no/low expression of PDL-1. Being cemiplimab not yet available, we resorted to cetuximab, the use of which is off-label for cSCC. We administered cetuximab at an initial single dose of 400mg/m2, followed by 250mg/m2 every week, for seven cycles, and every two weeks, for 35 total cycles. Follow-up assessment with periodic CT at three ( Figure 2b and 2c) and six months documented stable locally advanced disease. Therapy was well tolerated, with the only complaint of an acneiform eruption, which began after one week of treatment and was managed with clindamycin 1% gel twice a day and oral minocycline 100mg twice a day for four weeks.

The patient was restaged after 44 weeks with a whole-body CT scan that demonstrated progression to stage IV disease (T3N2bM1) with metastatic involvement. Combination therapy with the addition of PD-1 blocker was planned and we employed locally available anti-PD1 monoclonal antibody nivolumab according to the following scheme: cetuximab single dose of 250mg/m2 Q2W and nivolumab single fixed dose of 240mg Q2W administered at alternating weeks. Sequential CT assessments after 12 and 26 weeks showed stable disease at best, with slight increase of the primitive lesion and unchanged nodal and metastatic localizations.

## Discussion

In our report, response to cetuximab as a single agent and in combination with nivolumab was assessed for as long as 80 weeks.

We were challenged to select an effective treatment in this advanced case and resorted to EGFR inhibitor therapy. Cetuximab is approved for the treatment of locally or regional advanced SCC of the head and neck region (in combination with radiation) or for recurrent or metastatic disease (alone or in association with platinum). Its use in cSCC of other regions is currently off-label but our choice of drug was extensively supported by evidence in published literature ^3^. A phase II study of unresectable cSCC treated with cetuximab for at least six weeks registered 25% objective response and 42% disease stabilization ^6^. A diffuse papulopustular acneiform eruption is the most common cutaneous reaction pattern to EGFR inhibitors, reported in over two-thirds of treated subjects but severe in only 5–10% of cases. Cutaneous toxicity is suggested to be a proxy for response to cetuximab ^7^.

Immune checkpoint inhibition revolutionized the management of advanced cSCC and anti-PD-1 monoclonal antibody cemiplimab is currently the preferred first line therapy, following registration for this specific indication in the US in July 2018 and in Europe in July 2019. Response to cemiplimab was reported in 50% of patients in the expansion cohorts of the phase 1 study and in 47% of patients in the metastatic-disease cohort of the phase 2 study, with response exceeding 6 months in 57% of cases ^4, 8^.

Nivolumab monotherapy is indicated for the treatment of recurrent or metastatic SCC of the head and neck in adults progressing on or after platinum-based therapy. In a patient without access to cemiplimab clinical trials, nivolumab was our agent of choice due to the biological similarity to SCC of the head-neck district ^9^.

Recent experience from the literature attributes long-term remission and good tolerability to PD-1 checkpoint inhibition with nivolumab in cSCC. A series of three patients with advanced cSCC treated with nivolumab reported partial response in two subjects and stable disease in the third ^10^.

Chen *et al.* recently reported a case of clinical regression of invasive cSCC after six months of dual treatment with cetuximab weekly and nivolumab biweekly and hypothesized the mechanisms underlying a synergistic action of these two agents ^11^.

## Conclusions

- Serial biopsies are mandatory for advanced BCC candidates prior to vismodegib treatment to exclude foci of multiple differentiation ^12^.
- Prior to the introduction of cemiplimab, no drugs were approved specifically for cSCC.
- The efficacy of cetuximab is limited as a single agent, with modest durations for stable disease.
- Low PDL-1 expression does not preclude the efficacy of checkpoint inhibitors; in fact, cemiplimab is approved without requirement for testing.
- PD-1 blockade is the new standard of care in advanced cSCC in immunocompetent patients.

## Data availability

All data underlying the results are available as part of the article and no additional source data are required.

## Consent

Written informed consent for publication of their clinical details and clinical images was obtained from the patient.
