# Supplementary material for: Case Report: Cetuximab and nivolumab use in advanced cutaneous squamous cell carcinoma resistant to chemotherapy
Source: F1000Res. 2019 Dec 31;8:933. Originally published 2019 Jun 21. [Version 2] doi: 10.12688/f1000research.19149.2 (PMC6993817; doi:10.12688/f1000research.19149.2)
Supplement: Peer review history

# Peer review: Reviewer response for version 2

**DOI:** 10.5256/f1000research.23781.r58223

**Published:** 2020-01-27

**Authors:** Gloria Orlando

**Affiliations:** 1 Department of Medicine, Unit of Dermatology, University of Padova, Padua, Italy

This is a well written and interesting paper describing the effective use of cetuximab and nivolumab in an extensive thoracic cutaneous squamous cell carcinoma resistant to all previous lines of chemotherapy.

 The authors provide a detailed description of the case with its findings comprehensively summarized in the Table. 

 I think it is a useful publication describing current therapeutic strategies for NMSC and encouraging readership to perform serial biopsies in skin cancers where collision tumours may be present.

I confirm that I have read this submission and believe that I have an appropriate level of expertise to confirm that it is of an acceptable scientific standard.

# Peer review: Reviewer response for version 2

**DOI:** 10.5256/f1000research.23781.r58066

**Published:** 2020-01-07

**Authors:** Gregory A Daniels

**Affiliations:** 1 Department of Medicine, Division of Hematology/Oncology, University of California San Diego, La Jolla, CA, USA

No further comments.

I confirm that I have read this submission and believe that I have an appropriate level of expertise to confirm that it is of an acceptable scientific standard.

# Peer review: Reviewer response for version 1

**DOI:** 10.5256/f1000research.20985.r53108

**Published:** 2019-09-03

**Authors:** Gregory A Daniels

**Affiliations:** 1 Department of Medicine, Division of Hematology/Oncology, University of California San Diego, La Jolla, CA, USA

The case of this 60 y/o man with a history of at least two non-melanoma skin cancers details the use of targeted hedgehog inhibitors for the initial BCC histology followed by cytotoxic chemotherapy and ultimately EGFR directed treatment for what now appears to be cuSCC at the same location. The authors raise the important point of re-biopsy at progression particularly in skin cancers where blended histologies or collision tumors may occur.

 The use of Cetuximab has been a strategy for some time. The results outlined here are consistent with what is known—relatively modest clinical value. As referenced, the largest series found a relatively low response rate with modest durations for stable disease. The follow up here is short and the three month assessment is stable disease at best. Thus, the use of Cetuximab is of limited benefit as a single agent.

 Anti-PD1 therapy is referenced as an option at progression. For usual advanced cuSCC in elderly not immune suppressed patients, this is actually the preferred first line therapy. The monoclonal Cemiplimab was approved for use in Europe in July of 2019 and prior to that in the US following the NEJM publication by Migden et al July 2018. ^1^ The field has dramatically changed and the case should be updated to reflect this change. In the absence of a contra-indication of immune therapy, anti-PD1 therapy is standard of care.

 It would be very interesting to see an update to this case and the progress of this patient. At that point, the case would provide more information to clinicians.

 Specific suggestions:

1. Include the NEJM paper outlining use of Cemiplimab in cuSCC not amenable to curative surgery or radiation.
2. Provide longer follow up than the 12 weeks reported.
3. Outline if the patient was treated with anti-PD1 therapy and why not.
4. Comment on toxicity of therapies.
5. The authors are correct in making the statement that PDL1 testing may not be needed for response to anti-PD1 therapy in this disease. In fact, Cemiplimab is approved without the requirement for testing.
6. If available, it would be interesting to see NGS on the tumor. The tumor mutation burden for cuSCC is very high with some usual mutations (i.e. p53 and NOTCH). As this was a confusion in the case, this data may help clarify the origin of this tumor.

I confirm that I have read this submission and believe that I have an appropriate level of expertise to confirm that it is of an acceptable scientific standard, however I have significant reservations, as outlined above.

## References

[1] : PD-1 Blockade with Cemiplimab in Advanced Cutaneous Squamous-Cell Carcinoma. *New England Journal of Medicine*.2018;379(4) : 10.1056/NEJMoa1805131 341-351 10.1056/NEJMoa1805131 29863979

# Response

**Published:** 2019-12-03

**Authors:** Alvise Sernicola

**Affiliations:** Sapienza University of Rome, Italy

**Dear reviewer,**

**Thank you for your very accurate review of our paper. We have carefully reviewed all of your suggestions and corrections and revised the manuscript accordingly. Our responses are given in a point-by-point manner below.**

**We hope the revised version is now suitable for publication and look forward to hearing from you.**

****

Specific suggestions:

1.              Include the NEJM paper outlining use of Cemiplimab in cuSCC not amenable to curative surgery or radiation.

**Reference to the paper by Migden was added to the introduction: “In 2018, a study by Migden et al. dramatically changed the previous scenario establishing the new standard of care with PD-1 blockade in immune-competent patients, in the absence of contra-indications to immunotherapy (4). Anti-PD1 monoclonal antibody cemiplimab was consequently approved for use in Europe in July 2019.”**

2.              Provide longer follow up than the 12 weeks reported.

**We have provided a follow up of up to 80 weeks of treatment with cetuximab as a single agent (54 weeks) and in combination with nivolumab (26 weeks).**

3.              Outline if the patient was treated with anti-PD1 therapy and why not.

**Following progression to stage IV disease during cetuximab therapy, “Combination therapy with the addition of PD-1 blocker was planned and we employed locally available anti-PD1 monoclonal antibody nivolumab according to the following scheme: cetuximab single dose of 250mg/m2 Q2W and nivolumab single fixed dose of 240mg Q2W administered at alternating weeks.” (case presentation)**

4.              Comment on toxicity of therapies.

**Adverse events to cetuximab has been added to the case presentation: “Therapy was well tolerated, with the only complaint of an acneiform eruption, which began after one week of treatment and was managed with clindamycin 1% gel twice a day and oral minocycline 100mg twice a day for four weeks.”**

**Toxicity has been commented in the discussion: “A diffuse papulopustular acneiform eruption is the most common cutaneous reaction pattern to EGFR inhibitors, reported in over two-thirds of treated subjects but severe in only 5-10% of cases. Cetuximab cutaneous toxicity is suggested to be a proxy for treatment response (10).”**

5.              The authors are correct in making the statement that PDL1 testing may not be needed for response to anti-PD1 therapy in this disease. In fact, Cemiplimab is approved without the requirement for testing.

**Thank you, this has been made explicit in the conclusions: “Low PDL-1 expression does not preclude the efficacy of checkpoint inhibitors; in fact, cemiplimab is approved without requirement for testing.”**

6.              If available, it would be interesting to see NGS on the tumor. The tumor mutation burden for cuSCC is very high with some usual mutations (i.e. p53 and NOTCH). As this was a confusion in the case, this data may help clarify the origin of this tumor.

**Thank you for your suggestion, NGS assessment of tumor mutation burden was not available.**
